# Headache, migraine and risk of brain tumors in women: prospective cohort study

**DOI:** 10.1186/s10194-015-0501-0

**Published:** 2015-03-01

**Authors:** Tobias Kurth, Julie E Buring, Pamela M Rist

**Affiliations:** 1 Division of Preventive Medicine, Department of Medicine, Brigham and Women’s Hospital, Harvard Medical School, Boston, USA; 2 The Department of Epidemiology, Harvard T.H. Chan School of Public Health, Boston, USA; 3 Inserm Research Center for Epidemiology and Biostatistics (U897) - Team Neuroepidemiology, University of Bordeaux, College of Health Sciences, 146 rue Léo Saignat - CS61292, 33076 Bordeaux, France

**Keywords:** Migraine, Headache, Brain tumor, Epidemiology, Women

## Abstract

**Background:**

While headache is a common symptom among brain tumors patients, often patients with common headache have concerns of being at risk for developing brain tumors. We aimed to disprove that migraine or headache in general is associated with increased risk of developing brain tumors.

**Methods:**

Prospective study among 39,534 middle-aged women, free of any cancer, and who provided information on headache history at baseline. We followed participants for occurrence of medical record-confirmed brain tumors. We ran multivariable-adjusted Cox proportional hazards models to evaluate associations between any headache, migraine, and non-migraine headache with incident brain tumors. We further evaluated whether migraine frequency and updated headache information during follow-up could be linked with brain tumors.

**Results:**

A total of 13,022 (32.9%) women reported headache, of which 5,731 were classified as non-migraine headache and 7,291 as migraine. During a mean follow-up of 15.8 years, 52 brain tumors were confirmed. The multivariable-adjusted hazard ratios (95% confidence interval) for brain tumors were 1.33 (0.76-2.34) for any headache, 1.18 (0.58-2.41) for migraine and 1.53 (0.75-3.12) for non-migraine headache. The association for any headache was further attenuated in time-varying analyses (1.15; 0.58-2.24). Those who experience migraine six times/year were also not at increased risk of brain tumor (0.67; 0.13-3.32).

**Conclusions:**

Results of this large, prospective cohort study in women do not provide evidence that headache in general or migraine in particular are associated with the occurrence of brain tumors. Our data should reassure patients with headache that brain tumor is not a long-term consequence of headache.

## Background

Headache in general and migraine in particular are very common disorders on the population level. While common headaches with typical pain characteristics are not considered a risk factor for the development of brain tumors, patients who experience frequent or severe headache often worry about the possibility of a more serious underlying condition such as a life-threatening brain tumor [1,2].

Headache is a common symptom of brain tumors. In a series of 206 patients with brain tumors, 48% presented with headache [3]. Risk factor for presenting with headache were female gender, younger age, and a history of headache. In particular of the brain tumor patients with longstanding primary headache disorder history, 64% presented with headache [3]. While numerous articles report on specifics of headache as a symptom of brain tumors [4-6], there is a lack of data on the association of headache and migraine on the development of brain tumors.

Thus, we aimed to provide evidence that headache in general and migraine in particular are not associated with the development of brain tumors. To study our aims, we utilized data from the prospective Women’s Health Study which collected information on headache and migraine at baseline and confirmed brain tumor cases during a mean of 15.8 years of follow-up.

## Methods

### Study population

The Women’s Health Study was a randomized double blind placebo controlled trial to test the risks and benefits of low dose aspirin and vitamin E in the primary prevention of cardiovascular disease and cancer among apparently healthy women. The design, methods, and results have been described in detail previously [7-9]. Briefly, a total of 39,876 US female health care professionals aged 45 years or older at study entry (1992–1995) without a history of cardiovascular disease, cancer, or other major illnesses were randomly assigned to receive active aspirin (100 mg on alternate days), active vitamin E (600 IU on alternate days), both active agents, or both placebos. All participants provided written informed consent, and the institutional review board of Brigham and Women’s Hospital, Boston, MA, approved the WHS. Baseline information was self-reported and collected by a mailed questionnaire that asked about several cardiovascular risk factors and lifestyle variables. Twice in the first year and yearly thereafter, participants were sent follow-up questionnaires asking about study outcomes and other information during the study period. The trial ended in March 2004. At this time point, 89% of the initially randomized women who were still alive were eligible and willing to enter observational follow-up. For the purpose of this analysis, we included information from the time of randomization through October 2013.

### Assessment of headache and migraine

At baseline the women were asked “Have you ever had migraine headaches?” and “In the past year, have you had migraine headaches?” Women who experienced migraine headaches within the past year were asked for further details including “What is the approximate frequency of your migraines?” with possible response options of daily, weekly, monthly, every other month, and less than 6 times per year and “Do your migraines have any of the following characteristics?” The response options included “Aura or any other indication a migraine is coming” which was used to classify women who experience migraine with aura. Since the baseline questionnaire did not ask about non-migraine headache, we used information from the six-month questionnaire to assess headache. On the six-month questionnaire, women were asked if they had any headache since the baseline questionnaire. Using the women’s responses to these questions, we divided them into the following groups: any history of migraine (women who reported ever experiencing migraine headache), history of non-migraine headache (women who reported experiencing headache but did not report migraine headaches) and no history of headache (women who reported no history of migraine headache or non-migraine headache). We also created an “any headache” category which included the women in both the any history of migraine category and the history of non-migraine headache category. Women with any history of migraine were further divided into the following categories: “migraine with aura” (women who reported migraine within the past year and who reported the presence of aura or any indication that migraine is coming); “migraine without aura”; and “past history of migraine” (women who reported ever having migraines but not within the past year). Women who reported experiencing migraine within the past year were also categorized according to the frequency of their attacks (<6 times per year versus ≥6 time per year). A previous study in the Women’s Health Study [10] has shown good agreement between self-reported migraine without aura and classification of migraine without aura based on the International Classification of Headache Disorders criteria [11]. In particular, we have shown that 87% of women who reported migraine could be classified as “probable migraine” [10].

### Brain tumor assessment

Participants were asked to report tumor diagnoses on each follow-up questionnaire. Medical records and other relevant information were obtained for all self-reported brain tumor cases and reviewed by an Endpoints Committee of physicians to confirm the medical diagnosis. Deaths of participants were identified through family member reports, postal authorities, or a search of the National Death Index. For the purpose of this analysis, only confirmed first primary brain tumor events were included.

### Statistical methods

Of the 39,876 participants, 119 women with missing migraine information at baseline were excluded. We additionally excluded 223 women who developed any tumor prior to the 6-month questionnaire or did not return the six-month questionnaire, leaving a total of 39,534 women free of any tumor for this analysis.

Baseline characteristics of participants according to headache and migraine status were compared by contrasting means or frequencies.

Person-time was calculated from the date of the 6-month questionnaire to the date of first brain tumor diagnosis, date of first any other tumor diagnosis, death from any cause, last contact, or end of follow-up, whatever occurred first.

We used Cox proportional hazards models to evaluate the association of any headache, non-migraine headache, and any history of migraine on brain tumor incidence. We calculated age-and multivariable-adjusted hazard ratios (HRs) and their corresponding 95% confidence intervals (CIs).

To determine whether the risk for developing brain tumor differs according to migraine subtypes, we calculated age-adjusted HRs (95% CI) for migraine subtypes (migraine with aura, migraine without aura, or past history of migraine) and brain tumor.

We also performed exploratory analyses in which we ran multivariable-adjusted models stratified by age (<55 years of age compared to ≥55 years of age).

To explore whether migraine attack frequency is associated with brain tumor risk, we calculated age-adjusted HRs for any brain tumor according to the reported number of migraine attacks among the 5,081 active migraineurs who provided attack frequency information.

The multivariable-adjusted models were adjusted for age, body mass index (BMI) (<25, 25–29.9, ≥30 kg/m^2^), alcohol consumption (rarely/never, 1–3 drinks/month, 1–6 drinks/week, ≥1 drink/day), smoking status (never, past, current), postmenopausal status (premenopausal, postmenopausal, uncertain), and exercise (rarely/never, <1 time/week, 1–3 times/week, ≥4 times/week). Additional adjustment for randomized treatment assignments did not change the effect estimates of migraine and non-migraine headache on any brain tumor.

Less than 100 people had missing information on any of our covariates. Those with missing information on alcohol consumption or exercise were assigned to the reference category (rarely/never). Those with missing information on smoking were assigned to the past smoking category and those missing information on postmenopausal status were assigned to the uncertain category. Women missing BMI were assigned the mean BMI (26.1 kg/m^2^).

In secondary analyses, we updated the information on any headache during follow-up. On each follow-up questionnaire, participants were asked about headache or migraine. Those who did not report headache or migraine in the past were re-categorized to any headache if they indicated headache or migraine during follow-up.

The proportional hazards assumption was tested by including an interaction term for headache status and logarithm of follow-up time for any brain tumor in age-adjusted models. We found no statistically significant violation.

For all analyses, we used SAS (version 9.3, SAS Institute Inc. Cary, NC). All p-values were 2-tailed and p-value <0.05 was considered statistically significant.

## Results

Of the 39,534 participants, 13,022 (32.9%) women reported headache, of which 5,731 were classified as non-migraine headache and 7,291 as migraine headache.

In Table 1, baseline characteristics of participants according to headache status are presented. Those who experience migraine or non-migraine headache were younger, more likely to be never smokers and to rarely consume alcohol, and less likely to exercise frequently or be postmenopausal than those without headache.

**Table 1:** **Baseline characteristics according to headache status (N = 39,534)**

	**No headache (N = 26,512)**	**Non-migraine headache (N = 5,731)**	**Any migraine (N = 7,291)**
Age, yrs, mean	55.1 (7.3)	53.6 (6.4)	53.5 (6.4)
Body mass index, kg/m^2^, mean	25.9 (5.0)	26.3 (5.3)	26.2 (5.1)
Smoking, %			
Never	50.4	51.5	53.4
Past	36.2	36.0	34.0
Current	13.2	12.3	12.4
Alcohol consumption, %			
Rarely never	43.6	48.2	47.8
1-3 drinks/month	12.8	13.3	14.4
1-6 drinks/week	32.3	30.4	29.7
≥1 drinks/day	11.2	8.1	8.1
Exercise, %			
Rarely/never	38.4	37.7	38.7
<1/week	19.1	20.7	22.2
1-3 times/week	31.2	32.7	29.6
≥4 times/week	11.3	8.9	9.5
Postmenopausal status, %			
Premenopausal	26.9	30.1	28.0
Postmenopausal	56.4	49.7	50.2
Unsure	16.6	20.0	21.6

During a mean follow-up time of 15.8 years, 52 brain tumor cases were confirmed. There were 36 gliomas or glioblastomas, 7 astrocytomas, 3 meningiomas, 1 oligodendroglioma, and 5 other neoplasms. In Table 2, age- and multivariable-adjusted HRs (95% CI) for brain tumor according to headache status are presented. Those who experience any headache had a hazard ratio of 1.33 (95% CI: 0.76, 2.34) compared to those with no headache after multivariable adjustment. We also did not observe a significant increase in the risk of brain tumor when we examined those with non-migraine headache (HR = 1.53; 95% CI: 0.75, 3.12) or any migraine (HR = 1.18; 95% CI: 0.58, 2.41). The association for any headache was further attenuated in the time-varying analysis (1.15; 0.59-2.24). Stratification by age demonstrated a slightly higher risk of brain tumor among those less than 55 years of age, but none of the increases were statistically significant (Table 3).

**Table 2:** Age- and multivariable* adjusted hazard ratios (95% confidence intervals) of brain tumor according to headache status in the Women’s Health Study (N = 39,534)

		**No headache (n = 26,512)**		**Any headache (n = 13,022)**		**Non-migraine headache (n = 5,731)**		**Any migraine (n = 7,291)**
	**No. of cases**	**Hazard ratio (95% CI)**	**No. of cases**	**Hazard ratio (95% CI)**	**No. of cases**	**Hazard ratio (95% CI)**	**No. of cases**	**Hazard ratio (95% CI)**
Age-adjusted	32	1.00 (ref)	20	1.35 (0.77, 2.37)	10	1.53 (0.75, 3.13)	10	1.21 (0.59, 2.47)
Multivariable adjusted*	32	1.00 (ref)	20	1.33 (0.76, 2.34)	10	1.53 (0.75, 3.12)	10	1.18 (0.58, 2.41)

*Adjusted for age, body mass index (BMI) (<25, 25–29.9, ≥30 kg/m^2^), alcohol consumption (rarely/never, 1–3 drinks/months, 1–6 drinks/week, ≥1 drink/day), smoking status (never, past, current), postmenopausal status (premenopausal, postmenopausal, uncertain), and exercise (rarely/never, <1 time/week, 1–3 times/week, ≥4 times/week).

**Table 3:** Multivariable* adjusted hazard ratios (95% confidence intervals) of brain tumor stratified by age according to headache status in the Women’s Health Study (N = 39,534)

		**No headache (n = 26,512)**		**Any headache (n = 13,022)**		**Non-migraine headache (n = 5,731)**		**Any migraine (n = 7,291)**
	**No. of cases**	Hazard ratio* (95% CI)	**No. of cases**	Hazard ratio* (95% CI)	**No. of cases**	Hazard ratio* (95% CI)	**No. of cases**	Hazard ratio* (95% CI)
<55 years of age at baseline (N = 23,849)	13	1.00 (ref)	12	1.57 (0.72, 3.46)	6	1.79 (0.68, 4.72)	6	1.40 (0.53, 3.69)
≥55 years of age at baseline (N = 15,685)	19	1.00 (ref)	8	1.10 (0.48, 2.53)	4	1.28 (0.43, 3.78)	4	0.96 (0.33, 2.85)

*Adjusted for age, body mass index (BMI) (<25, 25–29.9, ≥30 kg/m^2^), alcohol consumption (rarely/never, 1–3 drinks/months, 1–6 drinks/week, ≥1 drink/day), smoking status (never, past, current), postmenopausal status (premenopausal, postmenopausal, uncertain), and exercise (rarely/never, <1 time/week, 1–3 times/week, ≥4 times/week).

We also explored whether the risk of brain tumor varies by migraine subtype in age-adjusted analyses. We observed 2 cases of brain tumor in those with a past history of migraine (N = 2,145), 7 cases in those with migraine without aura (N = 3,096) and 1 case in those with migraine with aura (N = 2,049). Past history of migraine (HR = 0.70, 95% CI: 0.17, 2.88), migraine without aura (HR = 1.88, 95% CI: 0.84, 4.23), and migraine with aura (HR = 0.41, 95% CI: 0.06, 2.97) were not associated with a significant increase in the risk of brain tumor in age-adjusted models.

Of those who experienced active migraine at baseline, 3,293 women experienced migraines less than six times per year of which 6 developed brain tumor and 1,788 women experienced migraine six or more times per year of which 2 developed brain tumor. Those who experience migraine six or more times per year were not at increased risk of brain tumor compared to those who experience migraine less than six times per year (HR = 0.67, 95% CI: 0.13, 3.32) in age-adjusted models.

## Discussion

In this large, prospective study of initially apparently healthy women, we did not find evidence that any headache history is associated with increased risk of brain tumors. The lack of association was apparent for women with any headache history, non-migraine headache, or migraine headache. The results were further attenuated when we updated information on any headache during follow-up. We also found no association for women who indicated they experienced more frequent migraine attacks. While numbers were low, we also did not find any evidence that migraine aura status was associated with increased risk of brain tumor incidence.

### Comparison with other studies

While there are numerous studies which report headache as a symptom of brain tumors [3,4,12,13], to the best of our knowledge, no other population-based study has evaluated the role of headache as risk factor for the development of brain tumor. A recent review of the literature summarized the evidence for headache as a symptom of brain tumors [13]. The authors found that the headaches associated with brain tumors often satisfy the criteria for a primary headache category such as migraine or tension-type headache but these headaches are often observed in patients with a longstanding history of primary headache disorders [3].

Despite the lack of scientific evidence, there are reports on webpages describing fears of brain tumor development for patients with headache disorders, specifically for patients with chronic headache forms [1,2]. We hope that the lack of association of headache or migraine with the development of brain cancer presented in this study will help to remove this fear for patients and contribute to a more rational discussion. Of course our data do not contradict the fact that headache is a symptom of brain tumors or that brain tumors can trigger headache disorders [13,14].

Several other studies have evaluated the association between migraine and overall cancer and breast cancer in particular. In a case–control study nested within the General Practice Research Database, Becker and colleagues found a slight increase in cancer risk (HR = 1.16; 95% CI: 1.09, 1.24) for patients with migraine [15]. While this association was adjusted for major other comorbid conditions and risk factors, increased healthcare utilization by patients with migraine may have accounted for this increased risk of cancer [15].

There is an ongoing discussion about whether patients with migraine are at decreased risk of developing breast cancer [16] based on the involvement of hormonal factors in migraine and breast cancer. However, recent population-based findings did not confirm a reduced breast cancer risk [17,18]. With regard to brain cancer, hormonal factors are very unlikely to play a role in the development of brain tumors [19]. We are also not aware of any other plausible biological mechanism that could link headache in general or migraine in particular with brain tumor development.

### Strengths and limitations

Our study has several strengths including its large size, standardized assessment of headache disorders at baseline, medical record confirmation of diagnoses of brain tumors, and the homogeneity of the cohort assuring similar access to medical care.

Several limitations have to be considered when interpreting our results. First, headache and migraine were self-reported and misclassification is possible. However, at least for migraine without aura, we have shown good agreement with standard diagnostic criteria [10]. In addition, migraine classification was done years before brain tumor diagnosis, which would result in non-differential bias. Second, although the Women’s Health Study is a large cohort, only few brain tumor cases occurred resulting in reduced power to detect a signal. Lastly, our study population consisted of female health professionals aged 45 years and older at baseline. Thus, our data may not generalize other settings. However, we have no reason to believe that the mechanisms by which headache potentially affects incident brain tumor differs in our study population.

### Clinical implications

We believe that the lack of association between any headache history and risk of developing brain tumor is of great importance for patients and their treating physicians. Our data should reassure patients that a primary headache disorder will not result in a brain tumor. Of course headache can be an early symptom of brain cancer but this secondary induced headache differs in symptoms and course from primary headache [14]. In our study, we did not study whether headache is a symptom reported by participants at the time of brain tumor incidence.

## Conclusions

In conclusion, results of our prospective cohort study do not indicate that headache in general or migraine in particular are risk factors for the development of brain tumors. We did not study headache as a symptom of brain tumors or whether brain tumors trigger specific headache forms. Patients with headache in general and migraine in particular should be reassured that brain tumor is very likely not a consequence of their disorder.
